# Trends in Private Equity Acquisitions of Assisted Living Facilities

**DOI:** 10.1001/jamanetworkopen.2025.43864

**Published:** 2025-11-17

**Authors:** Jennifer N. Bunker, Yashaswini Singh, Gauri Gadkari, John Bowblis, Lindsey Smith, Paula Carder, Sean Huang, Momotazur Rahman, Kali S. Thomas

**Affiliations:** 1 School of Nursing, Johns Hopkins University, Baltimore, Maryland; 2 School of Public Health, Brown University, Providence, Rhode Island; 3 Department of Economics, Farmer School of Business, Miami University, Oxford, Ohio; 4 Scripps Gerontology Center, Miami University, Oxford, Ohio; 5 OHSU-PSU School of Public Health, Portland, Oregon; 6 Institute on Aging, Portland State University, Portland, Oregon; 7 Department of Health Systems Administration, Georgetown University, Washington, DC

## Abstract

This cross-sectional study examines trends in private equity acquisitions of assisted living facilities across the US.

## Introduction

Assisted living (AL) is a widely used long-term care option, serving nearly 1 million older adults across more than 32 000 US facilities.^1^ While growing private equity (PE) investment in nursing homes raises concerns about staffing^2^ and quality of care,^3^ the role of PEs in AL is unknown. As demand for long-term care grows,^4^ understanding who is acquiring AL facilities is critical. This study examines trends in PE acquisitions of AL facilities.

## Methods

Per the Common Rule, this cross-sectional study did not require ethics review because it constituted non–human participant research. We followed the STROBE reporting guideline.

We used LevinAssociates’ LevinPro LTC acquisitions database to source AL acquisitions, relying on LevinAssociates’ identification of the majority or controlling buyer as PE (eMethods in Supplement 1). We defined AL as any state-licensed facility that houses older adults and provides at least 2 meals per day, 24-hour supervision, and assistance with activities of daily living.

We identified 3044 senior housing acquisitions through 2023, of which 320 involved PE and AL. We used a stepwise approach to remove duplications, supplement missing information, and match data to a national directory of licensed AL facilities for 2017 to 2023^5^ (eTable in Supplement 1). Nonmatching facilities that were acquired before the directory period were confirmed as AL using the Internet Archive. We report PE acquisition trends from 2006 to 2023, including counts, locations, and inflation-adjusted transaction values. Data were analyzed from January 8, 2024, to May 3, 2025.

## Results

**Figure 1.**
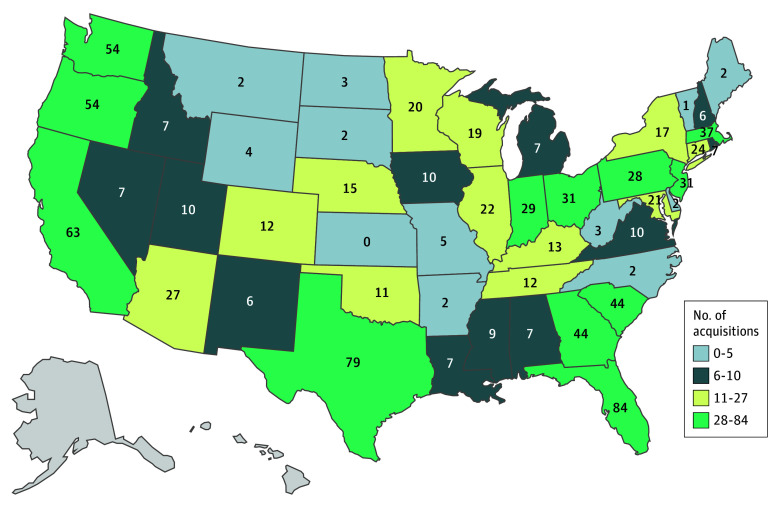
Number of Private Equity Acquisitions of Assisted Living Facilities by State

We identified 252 PE acquisitions involving 912 AL facilities. A majority of deals (172 [68.2%]) involved a single AL; only 20 acquisitions (7.9%) included more than 5 AL facilities. PE-acquired AL facilities were in 47 states, but more than one-third (n = 334 [36.6%]) were in Florida (n = 84), Texas (n = 79), California (n = 63), Washington (n = 54), and Oregon (n = 54) (Figure 1). The transaction dollar value was reported for 170 acquisitions (67.5%), representing 81.4% (n = 742) of all acquisitions. The total reported transaction value of AL acquisitions was $17.3 billion in inflation-adjusted 2023 dollars.

**Figure 2.**
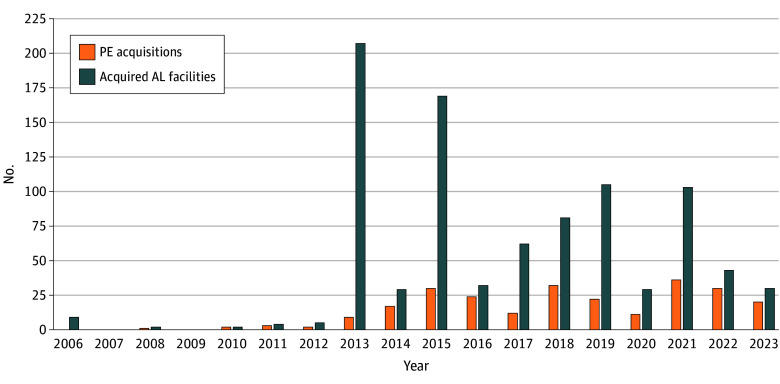
Number of Private Equity (PE) Acquisitions of Assisted Living (AL) Facilities and Number of Acquired Facilities by Year

Yearly PE acquisitions involving AL facilities were low from 2006 to 2012 but increased between 2013 and 2023 (Figure 2), with the highest adjusted values in 2019 ($2.84 billion). Total acquisitions were highest in 2021, with 36 transactions involving 103 facilities.

## Discussion

This cross-sectional study is, to our knowledge, the first to report trends in PE acquisition of AL facilities. The rise in acquisitions during the past decade parallels trends in other care settings and likely reflects anticipated growth in long-term care demand driven by the aging population.^4^ We provide new insights by addressing challenges related to reporting PE investment in AL facilities. Because states license, certify, and refer to AL differently, no public national list of facilities exists. Additionally, states may not require licensees to report ownership information. We addressed these challenges by compiling a national directory of licensed facilities^5^ via a uniform definition of AL applied across states^6^ and linking mergers and acquisitions data with other information sources to identify PE acquisitions of this growing long-term care setting.

Because documentation of PE acquisitions of AL facilities is opaque, these results likely underestimate the actual number of acquisitions. Our dataset did not include AL facilities developed with PE capital, acquisitions not captured by LevinAssociates, or transactions occurring before the database’s coverage; additionally, we could not determine the extent of PE influence on operations.

This study provides a foundation for further inquiry into PE in AL facilities. With the aging population driving demand for long-term care and the lack of public financing options for AL, future research should investigate the influence and nuances of PE involvement on access to AL and residents’ experiences.

## Notes

Supplement 1.
eMethods
**eTable.** Methodology for Refining Private Equity Acquisitions of Assisted Living Data

Supplement 2.
Data Sharing Statement
