# Supplementary material for: Trends in Private Equity Acquisitions of Assisted Living Facilities
Source: JAMA Netw Open. 2025 Nov 17;8(11):e2543864. doi: 10.1001/jamanetworkopen.2025.43864 (PMC12625683; doi:10.1001/jamanetworkopen.2025.43864)
Supplement: Supplement 1. — eMethods eTable. Methodology for Refining Private Equity Acquisitions of Assisted Living Data [file jamanetwopen-e2543864-s001.pdf]

Supplemental Online Content

Bunker JN, Singh Y, Gadkari G, et al. Trends in private equity acquisitions of assisted living facilities. *JAMA Netw Open*. 2025;8(11):e2543864. doi:10.1001/jamanetworkopen.2025.43864

eMethods
eTable. Methodology for Refining Private Equity Acquisitions of Assisted Living Data

This supplemental material has been provided by the authors to give readers additional information about their work.

© 2025 Bunker JN et al. *JAMA Network Open*.

eMethods

Methodology for identifying and refining private equity (PE) acquisitions of assisted living (AL) data.

We first reviewed LevinAssociates' LevinProLTC Database for all-time acquisitions considered "Senior Housing", resulting in 3,044 acquisitions. Among those acquisitions, we searched the transaction descriptions for "private equity" and for AL license terms, relying on (Step 2). We manually reviewed all 320 transactions for accuracy (Step 3); five team members searched additional sources (i.e., SEC filings, S&P's CapitalIQ, Pitchbook, internet archives, press/news releases, company websites, real estate records) to confirm acquired company or transaction details; we relied on LevinAssociates identification of an acquirer as PE and used these sources for additional details outside of PE identification. We excluded 68 transactions that were duplicates, not AL, or lacked sufficient information. Among 252 remaining transactions, we identified the number of AL facilities involved and the name and addresses of those facilities (Step 4). We attempted to match each facility to a national AL directory of licensed AL facilities between 2017 and 2023 (Step 5). For 70 non-matching facilities, we searched internet archives to determine whether facilities were AL at acquisition (Step 6).

It should be noted that LevinAssociates identifies an entity as "acquirer" when they are the majority or controlling buyer. Additionally, LevinAssociates reported that PE firms were identified based on self-reported classification in public sources; when unclear, additional verification was undertaken.

Search Terms Used to Identify Transactions Involving Assisted Living

ALF, Adult Care, Adult Family, Adult Foster, Adult Group Home, Adult Home, Adult Residential, Alternative Living Services, Assisted Care Living, Assisted Living, Basic Care, Boarding Home, CCRC, Care Facility, Care Home, Community-Based Residential Facility, Community Living, Community Residence Facility, Community Residential Care, Comprehensive Care Facility, Convalescent Home, Dementia Care, Elder Care, Elder Group Home, Elderly Housing, Enhanced Services Facility, Enriched Housing Program, Home Plus, Home for the Aged, Housing with Services, Long-Term Care Facility, Personal Care, Private Non-Medical Institution, RCF, RCFE, Rehabilitation Center, Residential Care, Residential Facility for Groups, Residential Health Care, Residential Home for the Aged, Residential Living Home, Respite Care, Rest Home, Retirement Home, Senior Living, Shared Housing, Shelter Care, Special Care Unit, Supported Living, Supportive Living.

© 2025 Bunker JN et al. *JAMA Network Open*.

eTable. Methodology for Refining Private Equity Acquisitions of Assisted Living Data

Step	Review Type	Resulting Data
Step 1	In the LevinPro LTC Database, identify all acquisitions classified as "Senior Housing"	3044 acquisitions
Step 2	Search 3044 acquisitions for terms related to private equity and assisted living (see Appendix Table 2 for complete list of search terms)	320 acquisitions potentially involving AL
Step 3	Transaction-level review<br>● Review 320 transaction details for AL relevance<br>● Review/supplement transaction details (i.e., value, close date)	252 acquisitions<br>● Excluded:<br>○ n=33 acquisitions that were duplicates<br>○ n=7 acquisitions without information<br>○ n=28 acquisitions with clear lack of AL involvement (e.g.; software companies)
Step 4	Facility-level review<br>● Review/supplement # of facilities involved in 252 acquisitions<br>● Review/supplement each facility name and location	252 acquisitions of 973 facilities
Step 5	Match to national AL directory<br>● Match 273 facility names and addresses to the national directory to validate that the facility was AL	903 AL facilities matched to national AL directory
Step 6	Manual review of 70 non-matched facilities	9 facilities were confirmed to be an AL facility<br>● Excluded:<br>○ n=26 independent living facilities<br>○ n=19 unknown facilities<br>○ n=7 nursing homes<br>○ n=2 apartments<br>○ n=2 development sites<br>○ n=2 medical offices<br>○ n=1 enhanced living<br>○ n=1 inpatient rehab facilities<br>○ n=1 long-term acute care
**Final Analytic Sample = N=252 acquisitions of 912 AL facilities**		

© 2025 Bunker JN et al. *JAMA Network Open*.
