# Supplementary material for: Trends in Private Equity Acquisitions of Assisted Living Facilities
Source: JAMA Netw Open. 2025 Nov 17;8(11):e2543864. doi: 10.1001/jamanetworkopen.2025.43864 (PMC12625683; doi:10.1001/jamanetworkopen.2025.43864)
Supplement: Supplement 2. — Data Sharing Statement [file jamanetwopen-e2543864-s002.pdf]

Data Sharing Statement

Bunker. Trends in Private Equity Acquisitions of Assisted Living Facilities. *JAMA Netw Open*.
Published November 17, 2025. doi:10.1001/jamanetworkopen.2025.43864

Data
Data available: No
